# Control of Neuronal Excitability by Calcium Binding Proteins: A New Mathematical Model for Striatal Fast-Spiking Interneurons

**DOI:** 10.3389/fnmol.2012.00078

**Published:** 2012-07-10

**Authors:** D. P. Bischop, D. Orduz, L. Lambot, S. N. Schiffmann, D. Gall

**Affiliations:** ^1^Laboratoire de Neurophysiologie, Faculté de Médecine, Université Libre de BruxellesBruxelles, Belgium

**Keywords:** calcium dynamics, parvalbumin, striatal fast-spiking interneurons, excitability, mathematical model

## Abstract

Calcium binding proteins, such as parvalbumin (PV), are abundantly expressed in distinctive patterns in the central nervous system but their physiological function remains poorly understood. Notably, at the level of the striatum, where PV is only expressed in the fast-spiking (FS) interneurons. FS interneurons form an inhibitory network modulating the output of the striatum by synchronizing medium-sized spiny neurons (MSN). So far the existing conductance-based computational models for FS neurons did not allow the study of the coupling between PV concentration and electrical activity. In the present paper, we propose a new mathematical model for the striatal FS interneurons that includes apamin-sensitive small conductance *Ca*^2+^-dependent *K*^+^ channels (SK) and the presence of a calcium buffer. Our results show that a variation in the concentration of PV can modulate substantially the intrinsic excitability of the FS interneurons and therefore may be involved in the information processing at the striatal level.

## Introduction

Calcium regulates many cellular processes, including hormone secretion, neurotransmitter release, ionic channel permeability, and gene transcription. The cytosolic calcium proteins are classified in trigger or buffer proteins (Schwaller, 2009). Trigger proteins, such as calmodulin, change their conformation upon binding *Ca*^2+^, as opposed to buffer proteins (e.g., calretinin, calbindin, or parvalbumin) which bind *Ca*^2+^ as its concentration increases within a cell and are thought to mainly act as passive modulators of the cytosolic calcium level. Nevertheless, it has been suggested that calbindin also acts as a *Ca*^2+^ sensor (Schmidt et al., 2005; Lambers et al., 2006). Moreover at the neuronal level, several results have shown that calcium buffers play a key functional role in the control of the neuronal firing. More precisely, it has been shown that the concentration of calretinin, acting as a fast calcium buffer, controls the excitability of cerebellar granule cells, through the activation of high-conductance voltage- and *Ca*^2+^-activated *K*^+^ (BK) channels (Gall et al., 2003, 2005; Bearzatto et al., 2006). Furthermore changes in the buffer concentration can dramatically affect the electrical discharge pattern of cerebellar granule cells, hence allowing transitions between regular firing and different types of bursting (Roussel et al., 2006).

PV is a member of the EF-hand calcium binding proteins family and it has two mixed *Ca*^2+^/*Mg*^2+^ binding sites. PV binds *Mg*^2+^ with medium affinity (*K_D,Mg_* ~ 5–500 μM) and *Ca*^2+^ with high affinity (*K_D,Ca_* ~ 5–100 nM; Schwaller, 2009). Under basal [*Ca*^2+^]*_i_* (100 nM), the majority of PV’s binding sites (>80%) are occupied by *Mg*^2+^. The binding of *Ca*^2+^ is determined by the slow *Mg*^2+^ off-rate (Schwaller, 2009). For this reason, PV is considered as a slow buffer similar to the synthetic chelator EGTA. However recent studies show that at high concentration and in certain physiological conditions, PV might also act as a fast buffer, similarly to the synthetic chelator BAPTA (Franconville et al., 2011; Eggermann and Jonas, 2012). In the striatum, PV is selectively expressed in the population of FS interneurons. Striatal FS interneurons exert a strong inhibitory control over MSN, the principal neurons of the striatum. FS interneurons can fire regular trains of action potentials (AP) at frequencies ranging from 20 to 200 Hz, with little spike-frequency adaptation. They can also exhibit stuttering firing patterns consisting of brief bursts of AP separated by quiescent periods, which are characterized by subthreshold membrane potential oscillations (Tepper et al., 2010). The FS interneuron firing patterns result from the expression of a specific set of voltage-gated channels (Zhang and McBain, 1995; Martina and Jonas, 1997; Erisir et al., 1999). For example, voltage-gated potassium channels of Kv3 type are responsible for the fast repolarization and short duration of AP (Rudy and McBain, 2001). We have strong indications for the presence of apamin-sensitive small conductance (SK) *Ca*^2+^-dependent *K*^+^ channels, that are known to be coupled to voltage-gated *Ca*^2+^ channels (Stocker, 2004). SK channels are voltage independent and are activated at free *Ca*^2+^ concentrations in the range of 300–700 nM (Hirschberg et al., 1998; Xia et al., 1998). In rat striatal fast-spiking interneurons, blockade of BK channels by iberiotoxin has no effect on action potential duration (Sciamanna and Wilson, 2011), hence SK channels are strong candidates for the observed spike-frequency adaptation in FS neurons (Maingret et al., 2008). The presence of this calcium-activated ionic conductance could provide a way for parvalbumin to control the discharge pattern of the FSI. In fact, in cerebellar granule cells, it has been shown that calretinin, a fast calcium buffer, modulates the excitability of cerebellar granule cells through the activation of BK channels (Gall et al., 2003).

In this study, we propose a new conductance-based computational model for striatal FS interneurons that includes the influence of PV and the presence of SK channels providing coupling between excitability and calcium dynamics during the spike generation. This model allows us to investigate the effect of variations in the concentration of PV on striatal FS interneurons activity. We show that excitability of FS neurons depends on PV concentration and that this regulatory effect occurs in a similar way for fast and slow buffers.

## Materials and Methods

### FS neuron computational model

Our computational model is adapted from the conductance-based model of Erisir (Erisir et al., 1999) of a FS neocortical interneuron. The ionic currents of the Erisir model consist of a fast transient Na^+^ current *I_Na_*, a fast delayed rectifier potassium current of Kv3.1 type *I_Kv3_*, a slow delayed rectifier potassium current of Kv1.3 type *I_Kv1_* and a passive leak current *I_leak_*. We add to this model a HVA calcium current *I_Ca_* (Stocker, 2004) and a SK potassium current, *I_SK_*. Charge conservation governs the membrane potential dynamics through the following equation:

(1) $$C_{m}\frac{dV}{dt}=-I_{Na}-I_{Kv1}-I_{Kv3}-I_{Ca}-I_{SK}-I_{leak}+I_{app}$$

where *C_m_*, *V* are the membrane capacitance and potential of the FS neuron, *I_app_* is an external applied current. The ionic currents are given by:

(2) $$I_{Na}=gN_{a}m_{\infty }^{3}h(V-V_{Na})$$

(3) $$I_{Kv1}=gK_{v1}n_{1}^{4}(V-V_{K})$$

(4) $$I_{Kv3}=gK_{v3}n_{3}^{2}(V-V_{K})$$

(5) $$I_{SK}=g_{SK}k^{2}(V-V_{K})$$

(6) $$I_{Ca}=g_{Ca}a_{\infty }^{2}(V-V_{Ca})$$

(7) $$I_{leak}=g_{leak}(V-V_{leak})$$

where *m* and *h* are respectively the activation and inactivation gating variables of the *I_Na_* current, *n*_1_, *n*_3_, *k*, *a* are respectively the activation variables of *I_Kv1_*, *I_Kv3_*, *I_SK_*, *I_Ca_* currents. The kinetic of the *m* and *a* activation variables are considered fast compared to the other gating variables and are set to their steady-state value *m* = *m*_∞_(*V*), *a* = *a*_∞_(*V*). The membrane capacitance was set to 30 pF, the leak conductance *g_leak_* to 2.5 nS and the leak reversal potential to −68 mV, to match the known experimental membrane capacitance (25–30 pF), membrane resistance (400 MΩ), and resting membrane potential (−70 mV). The other reversal potentials are *V_Na_* = 74 mV, *V_K_* = − 90 mV, *V_Ca_* = 80 mV, and the maximal ionic conductances are *g_Na_* = 700 nS, *g_Kv1_* = 2 nS, *g_Kv3_* = 300 nS, *g_Ca_* = 30 nS, *g_SK_* = 2 nS. The dynamic of the other gating variables (excepted *k*) are governed by:

(8) $$\frac{dx}{dt}=\alpha_{x}(V)(1-x)-\beta_{x}(V)x$$

(9) $$x_{\infty }=\frac{\alpha_{x}}{\alpha_{x}+\beta_{x}}$$

(10) $$\tau_{x}=\frac{1}{\alpha_{x}+\beta_{x}}$$

where *x* = *h*, *n*_1_, *n*_3_. The kinetics of the α*_x_*, β*_x_* are exactly as published in Mancilla et al. (2007). The kinetics of the *a* variable was adapted from Roussel et al. (2006) and follows:

(11) $$a_{\infty }=\frac{1}{1+\exp(\frac{-6-V}{7.775})}$$

(12) $$\tau_{a}=\frac{1}{\frac{8.0}{1+\exp(-0.072(V-5))}+\frac{0.1(V+8.9)}{\exp(0.2(V+8.9))-1}}$$

The *k* activation variable for SK channels is *Ca*^2+^ dependent and voltage independent. The equation for its time evolution was taken from Goldberg et al. (2009):

(13) $$\frac{dk}{dt}=\frac{\left(k_{\infty }({\left[Ca^{2+}\right]}_{i})-k\right)}{\tau_{k}}$$

(14) $$k_{\infty }=\frac{{\left[Ca^{2+}\right]}_{i}}{K_{SK}+{\left[Ca^{2+}\right]}_{i}}$$

(15) $$\tau_{k}=\frac{1}{K_{SK}+{\left[Ca^{2+}\right]}_{i}}$$

where *K_SK_* = *k_off,sk_*/*k_on,sk_*. The values of *k_on,sk_* = 0.4 μM^−1^ ms^−1^ (*Ca*^2+^-binding rate) and *k_off,sk_* = 0.2 ms^−1^ (from Goldberg et al., 2009). In presence of PV, the coupling of the calcium dynamic is done via the following equations:

(16) $$\frac{d{\left[Ca^{2+}\right]}_{i}}{dt}=\frac{I_{Ca}}{2FAd}-\gamma ({\left[Ca^{2+}\right]}_{i}-{\left[Ca^{2+}\right]}_{rest})-\frac{d{\left[PVC_{a}\right]}_{i}}{dt}$$

(17) $$\frac{d{\left[PVC_{a}\right]}_{i}}{dt}-k_{on,ca}{\left[Ca^{2+}\right]}_{i}{\left[PV\right]}_{i}-k_{off,ca}{\left[PVCa\right]}_{i}$$

(18) $$\frac{d{\left[PVMg\right]}_{i}}{dt}-k_{on,mg}{\left[Mg^{2+}\right]}_{i}{\left[PV\right]}_{i}-k_{off,mg}{\left[PVMg\right]}_{i}$$

where [*Ca*^2+^]*_i_* and [*PV*]*_i_* represent respectively the free intracellular *Ca*^2+^ concentration and the concentration of free PV. [*PVCa*]*_i_* and [*PVMg*]*_i_* are the concentration of PV bound to *Ca*^2+^ and *Mg*^2+^. The total PV concentration [*PV*]*_T_* = [*PV*]*_i_* + [*PVCa*]*_i_* + [*PVMg*]*_i_*. We assume that [*Mg*^2+^]*_i_* is constant as in (Lee et al., 2000). [*Mg*^2+^]*_i_* was set to 500 μM in agreement with the values found within neurons (300–600 μM; Li-Smerin et al., 2001). The association and dissociation constant of PV with *Ca*^2+^ and *Mg*^2+^ are *k_on,ca_* = 0.1 μM^−1^ ms^−1^, *k_off,ca_* = 0.001 ms^−1^, and *k_on,mg_* = 0.0008 μM^−1^ ms^−1^, *k_off,mg_* = 0.025 ms^−1^ (Lee et al., 2000). We consider *Ca*^2+^ fluxes across a shell of thickness *d* = 0.2 μm under cell surface (area *A* = 3000 μm^2^). The inward flux is −*I_Ca_*/2*FAd* (F is the Faraday constant). The term γ([*Ca*^2+^]*_i_* − [*Ca*^2+^]*_rest_*) is the clearance mechanism associated with the *Ca*^2+^ fluxes across the plasma membrane or storage organelles (γ = 1 ms^−1^, [*Ca*^2+^]*_rest_* = 0.07 μM). In presence of the slow or fast buffer, the coupling of the calcium dynamic is done via the following equations:

(19) $$\frac{d{\left[Ca^{2+}\right]}_{i}}{dt}=-\frac{I_{Ca}}{2FAd}-\gamma ({\left[Ca^{2+}\right]}_{i}-{\left[Ca^{2+}\right]}_{rest})-\frac{d{\left[BCa\right]}_{i}}{dt}$$

(20) $$\frac{d{\left[BCa\right]}_{i}}{dt}=k_{on}{\left[Ca^{2+}\right]}_{i}{\left[B\right]}_{i}-k_{off}{\left[BCa\right]}_{i}$$

where [*B*]*_i_*, [*BCa*]*_i_* are the concentration of free and bound buffer (slow or fast). The total buffer concentration [*B*]*_T_* = [*B*]*_i_* + [*BCa*]*_i_*. The list of model parameters are shown in Table 1. Numerical simulations traces are obtained after an initial integration of 4 s. The equations of the model are numerically solved using a fourth-order Runge–Kutta integration method (Press et al., 1992). The bifurcation diagram was built with the software XPPAUT 6.10 (Free Software Foundation Inc., Cambridge, USA).

**Table 1:** **Model parameters**.

Definition	Parameters	Values	Reference
Sodium conductance	*g_Na_*	700 nS	
Sodium reversal potential	*V_Na_*	74 mV	Jolivet et al. (2004)
*Kv*1 potassium conductance	*g_Kv1_*	2 nS	
*Kv*3 potassium conductance	*g_Kv3_*	300 nS	
SK potassium conductance	*g_SK_*	2 nS	
Potassium reversal potential	*V_K_*	−90 mV	Jolivet et al. (2004)
SK affinity for calcium	*K_SK_*	0.5 μM	Goldberg et al. (2009)
Calcium conductance	*g_Ca_*	30 nS	
Calcium reversal potential	*V_Ca_*	80 mV	Roussel et al. (2006)
Membrane capacitance	*C_m_*	30 pF	
Leak conductance	*g_leak_*	2.5 nS	
Leak reversal potential	*V_leak_*	−68 mV	
*Ca*^2+^ extrusion rate	γ	1 ms^−1^	
Shell thickness	*d*	0.2 μm	
*Mg*^2+^ concentration	[*Mg*^2+^]*_i_*	500 μM	Li-Smerin et al. (2001)
Resting *Ca*^2+^ concentration	[*Ca*^2+^]*_rest_*	0.07 μM	
Cell surface	*A*	3000 μm^2^	
*Ca*^2+^ binding rate to PV	*k_on,ca_*	0.1 μM^−1^ ms^−1^	Lee et al. (2000)
PV affinity for *Ca*^2+^	*K_D,ca_*	0.01 μM	Eberhard and Erne (1994)
*Mg*^2+^ unbinding rate from PV	*k_off,mg_*	0.025 ms^−1^	Lee et al. (2000)
PV affinity for *Mg*^2+^	*k_D,mg_*	31 μM	Eberhard and Erne (1994)
*Ca*^2+^ binding rate (fast buffer)	*k_on_*	0.1 μM^−1^ ms^−1^	Lee et al. (2000)
Affinity for *Ca*^2+^ (fast buffer)	*K_D_*	0.01 μM	Eberhard and Erne (1994)
*Ca*^2+^ binding (slow buffer)	*k_on_*	0.01 μM^−1^ ms^−1^	Schwaller et al. (2002)
Affinity for *Ca*^2+^ (slow buffer)	*K_D_*	0.1 μM	Schwaller et al. (2002)

## Results

### Experimental relevance of FS neuron computational model

We use a computational model, based on experimental data, to investigate how *Ca*^2+^ buffering by PV affects striatal FS interneuron excitability. Striatal FS neurons selectively express the slow calcium buffer PV (Kawaguchi et al., 1995). We have strong evidence that they also possess apamin-sensitive small conductance SK channels. Therefore we propose a FS interneuron model, adapted from Erisir et al. (1999), that includes the presence of SK channels and of PV calcium binding proteins. Since PV possesses mixed *Ca*^2+^/*Mg*^2+^ binding sites, we take into account in our model the competition between *Ca*^2+^ and *Mg*^2+^ for PV binding sites (see Materials and Methods). Until recently, parvalbumin was considered as a slow calcium buffer similar to the synthetic chelator EGTA (Schwaller, 2009) but new evidence shows that under certain physiological conditions, at high concentration, PV may act as a fast calcium buffer similar to the synthetic chelator BAPTA (Franconville et al., 2011; Eggermann and Jonas, 2012). Therefore we have included three buffering conditions in our study: PV with its mixed *Ca*^2+^/*Mg*^2+^ binding sites, a slow buffer similar to EGTA, and a fast buffer similar to the metal-free form PV. For these three buffering conditions, we have studied the effect of the calcium buffer on the excitability of FS neurons, for different buffer concentrations. In our model, the slow calcium buffer has a *K_D_* = 0.1 μM and a *k_on_* = 0.01 μM^−1^ ms^−1^ similar to EGTA (Schwaller et al., 2002; Schwaller, 2009). The fast calcium buffer has a *K_D_* = 0.01 μM and a *k_on_* = 0.1 μM^−1^ ms^−1^ similar to those of metal-free form PV (Eberhard and Erne, 1994; Lee et al., 2000).

**Figure 1:**
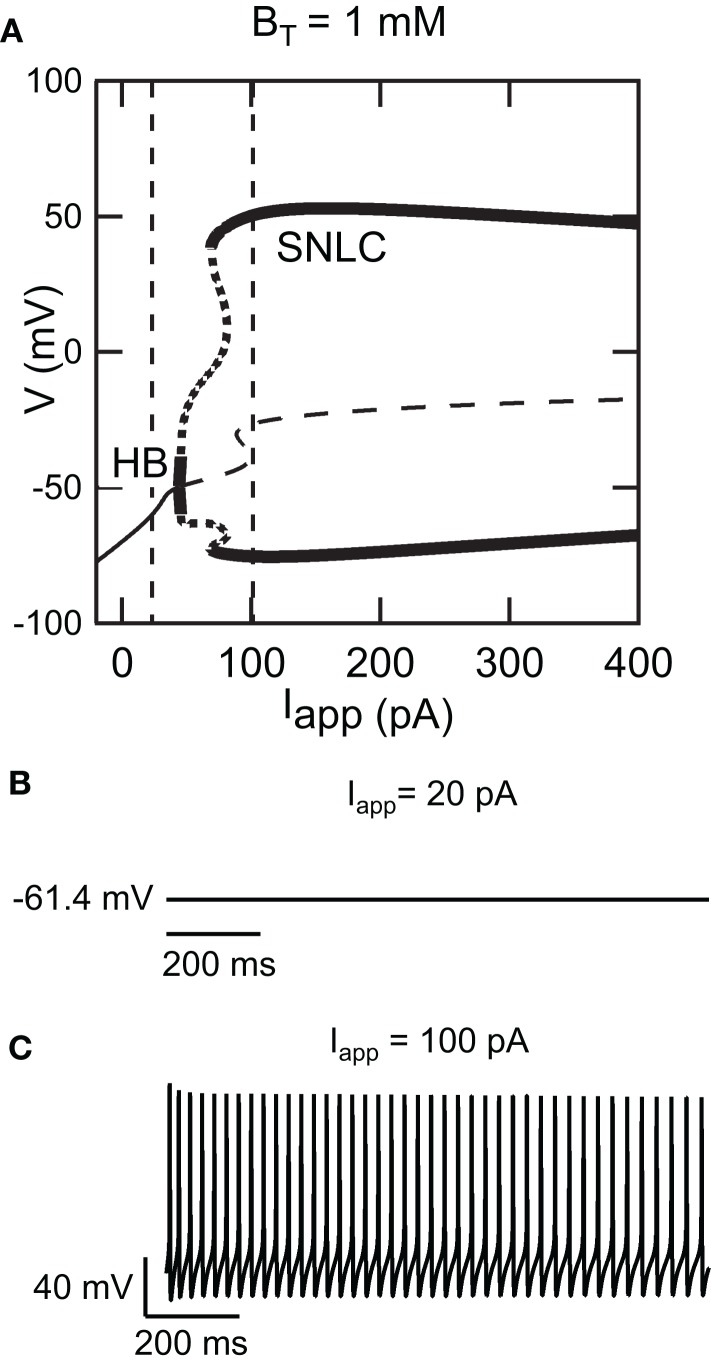
**Dynamical behavior of the FS interneuron computational model**. **(A)** Bifurcation diagram of our fast-spiking neuron model (*B_T_* = 1000 μM) representing voltage in function of applied current *I_app_*. As applied current increases from 0 pA, a branch of stable steady-states (thin solid curve) ends at a Hopf bifurcation (HB) point (*I_app_* ≈ 40 pA), giving rise to a branch of unstable periodic orbits (thick dotted line) and a branch of unstable steady-states at (thin dashed line) *I_app_* ≈ 40 pA. As the applied current increases further a branch of stable periodic solutions emerges from a saddle-node on a limit cycle (SNLC). Stable periodic solutions exist for *I_app_* > 69 pA. **(B)** Time series showing a stable steady-state solution at *I_app_* = 20 pA and **(C)** stable periodic solution at *I_app_* = 100 pA [the corresponding current values are displayed as dashed vertical lines in the bifurcation diagram shown in **(A)**].

**Figure 2:**
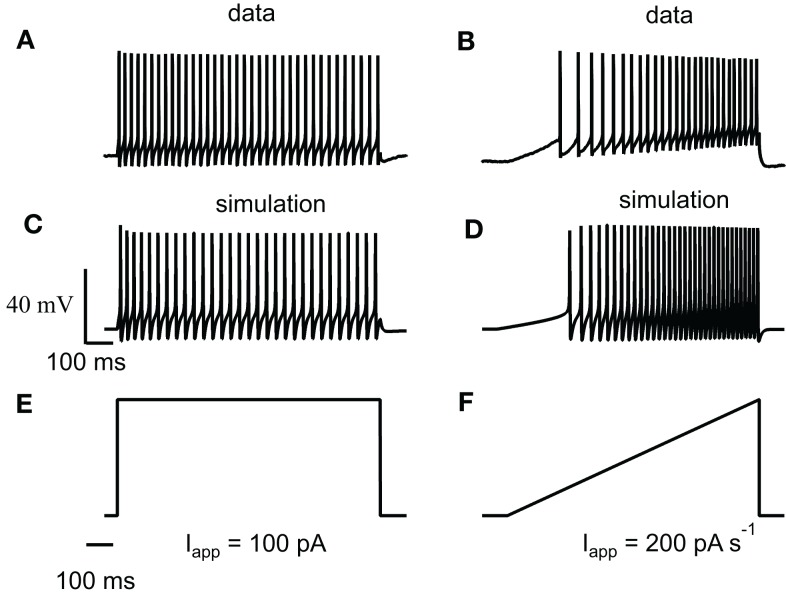
**Experimental relevance of the FS neuron computational model**. **(A)** Train of action potentials evoked in a striatal FS interneuron in response to a step and a ramp current injection during whole-cell recordings. During 1 s step of 100 pA the average spiking frequency is 36 Hz and the firing exhibits adaptation. **(B)** Whole-cell recording during a 1-s ramp of 200 pA s^−1^. Our model is adapted from Erisir et al. (1999) to include *Ca*^2+^, SK currents, and an endogenous calcium buffer. **(C,D)** Numerical simulations obtained with similar current protocols. The average spiking frequency in **(C)** is 31 Hz. The step and ramp current protocols used for the above experiment and simulation are shown in **(E,F)**. Experimental and simulated voltage traces exhibit similar firing patterns for both protocols demonstrating the quantitative aspect of the proposed computational model.

Figure 1A shows a bifurcation diagram of our FS model, with PV as calcium buffer, where the bifurcation parameter is the applied current (*I_app_*). Our model generates stable oscillations in the physiological range. A supercritical Hopf bifurcation (HB) at *I_app_* = 44 pA gives rise to a small window of periodic solutions that lose stability at *I_app_* = 45 pA. For *I_app_* > 44 pA there is a branch of unstable periodic solutions that ends at a saddle-node of limit cycle (SNLC), for *I_app_* = 68 pA. For *I_app_* = *I_SNLC_*, a branch of stable periodic solutions emerges. From that point the amplitude of the oscillations decreases as the applied current, *I_app_*, increases and the repetitive firing disappears at a supercritical Hopf bifurcation (not shown). A stable steady-state time series at *I_app_* = 20 pA and a stable periodic solution obtained at *I_app_* = 100 pA are shown in Figures 1B,C. Both the slow (similar to EGTA) and the fast (similar to the metal-free form PV) show a similar bifurcation diagram (data not shown). From a dynamical point of view, neurons are classified in two broad classes: class 1 and class 2 excitability (Izhikevich, 2007). Neurons in class 1 can fire at an arbitrary low frequency, depending on the strength of the applied current, while for neurons of class 2, the onset of oscillations starts at a non-zero frequency. Class 1 neurons can encode continuously the strength of an incoming stimulus in their firing frequency, while class 2 neurons will sense whether the strength of the stimulus is above a threshold. It has been shown experimentally that FS neurons share properties of class 2 neurons (Tateno et al., 2004). Accordingly, the electrical behavior of our FS model displays the typical dynamical behavior of class 2 neurons as the periodic firing originates from a Hopf bifurcation. In addition, typical experimental voltage traces obtained during whole-cell recording and the corresponding results of the numerical simulations are shown in Figure 2 for two different protocols: 1 s step current of 100 pA and 1 s ramp current injection of 200 pA s^−1^. In Figure 2A the FS interneuron fires at a typical frequency of 36 Hz whereas the result of the simulation gives a frequency of 31 Hz for the same protocol (1 s step current of 100 pA). Experimental and simulated voltage traces exhibit similar firing patterns for both protocols demonstrating the quantitative aspect of the proposed theoretical model.

### Summation of [*Ca*^2+^]*_i_* transients during trains of action potential

**Figure 3:**
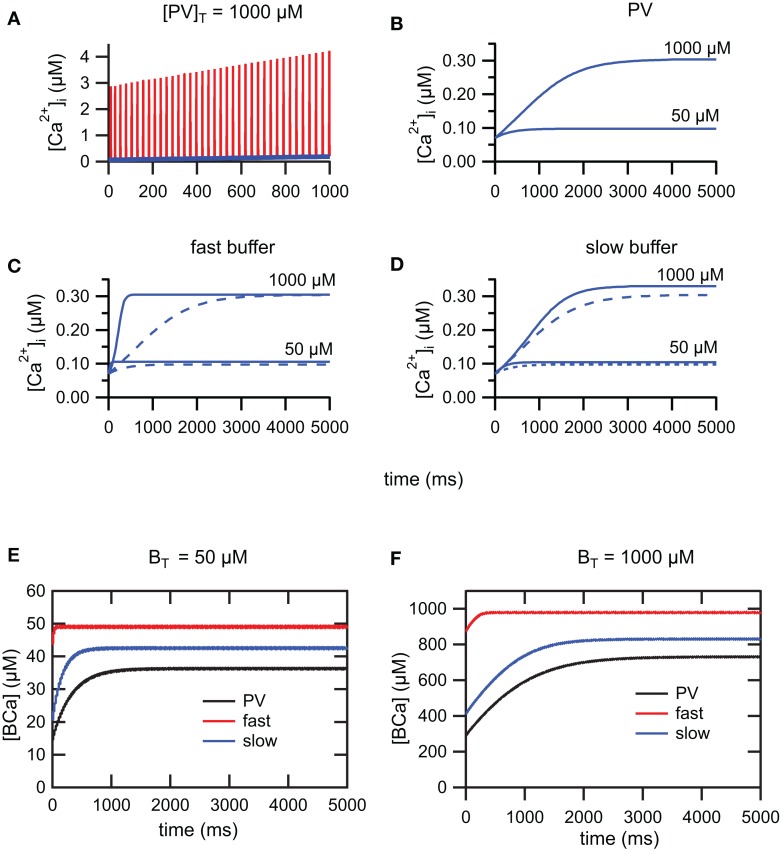
**Summation of [*Ca*^2+^]*_i_* transients during train of action potentials**. **(A)** Summation (red) and lower envelope (blue) of [*Ca*^2+^]*_i_* transients for the PV system (*I_app_* = 100 pA). **(B)** The time course of the lower envelope in the presence of PV. The time to reach the steady-state is delayed and the plateau level increases as the PV concentration increases. [*Ca*^2+^]*_i_* scales in **(A,B)** are different. **(C)** Due to saturation of the fast buffer, the time to reach the steady-state is faster for the fast buffer system (solid curve) compared to the PV system (dashed curve). Once PV is saturated with *Ca*^2+^, the PV system follows the time course of the fast buffer system. **(D)** For trains of action potentials of short duration, the time course of the PV system (dashed curve) follows the time course of the slow buffer system (solid curve). **(E,F)** Buffer occupancy vs. time. As for the *Ca*^2+^ transients, the buffer occupancy oscillates between lower and upper envelopes (not visible at this scale). The PV and slow buffer system will partially saturate after a few hundred milliseconds, whereas the fast buffer system is already saturated from early on. **(E)** *B_T_* = 50 μM and **(F)** *B_T_* = 1000 μM.

During a train of AP, [*Ca*^2+^]*_i_* increases due to the summation of [*Ca*^2+^]*_i_* transients. For trains of AP of sufficiently long duration, [*Ca*^2+^]*_i_* will reach a steady-state plateau level and fluctuate between a lower and upper level. At steady-state, calcium influx and clearance mechanisms compensate (Helmchen et al., 1996; Neher, 1998). During train of action potentials, evoked by 5 s depolarizing step current of 100 pA, we have investigated the time course to reach the steady-state using three buffering conditions. In the first condition, we have simulated PV with mixed *Ca*^2+^/*Mg*^2+^ binding sites. In the second and third conditions, we have simulated respectively a slow buffer similar to EGTA and a fast buffer similar to the metal-free form of PV. Figure 3A shows the summation and lower envelope of [*Ca*^2+^]*_i_* transients, in the presence of PV, during the first 1000 ms of a train of action potentials (*I_app_* = 100 pA). The time course of the lower envelopes of [*Ca*^2+^]*_i_* transients in the presence of PV is shown in Figure 3B. As previously demonstrated (Lee et al., 2000), the time to reach the steady-state is delayed and the plateau level increases as the PV concentration increases. A similar behavior is observed for the slow and fast buffer systems (Figures 3C,D). For the fast buffer the time to reach the steady-state is shorter than the time for the slow buffer (Figure 3C). Moreover, for trains of AP of short duration, the envelope of Ca^2+^ transients follows a similar time course both for PV and the slow buffer (Figure 3D). Whereas for trains of AP of longer duration, once PV is saturated with *Ca*^2+^, the lower envelope of *Ca*^2+^ transients follows a similar time course both for PV and the fast buffer (Figure 3C). In Figures 3E,F, the plots of the buffer occupancy show that PV and the slow buffer partially saturate after a few hundred of milliseconds, whereas the fast buffer system is already saturated at this time. The lower envelope of [*Ca*^2+^]*_i_* transients determines the *Ca*^2+^ available for the activation of SK channels during the interspike intervals and therefore the modulation of the firing frequency. In the following section, we study the effect of different calcium buffer concentrations on the excitability of FS neurons. Using 5 s step current of 100 pA, we only account for *Ca*^2+^ transients occurring at the steady-state plateau level during the 4th and 5th second of the train. The duration of the step current was chosen to allow *Ca*^2+^ transients and spike-frequency adaptation, occurring during trains of AP, to reach their steady-state.

### Regulation of FS excitability by fast and slow calcium buffers

**Figure 4:**
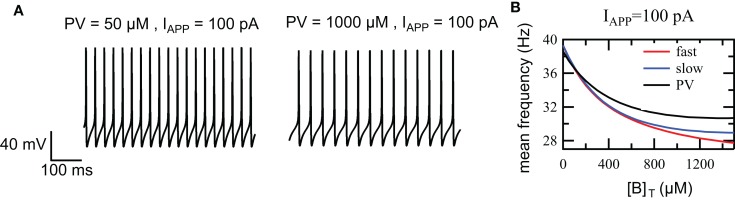
**Effect of the PV concentration on the intrinsic excitability in the FS cell model**. **(A)** The response of the FS cell model to injection of 100 pA depolarizing current for a buffer concentration *PV_T_* = 50 μM and *PV_T_* = 1000 μM. **(B)** Firing frequency vs. buffer concentration. The mean firing frequency decreases as the buffer concentration increases (PV: black, slow buffer: blue, fast buffer: red).

We have investigated the effect of different calcium buffer concentrations on FS excitability for PV, the slow and fast buffer systems. Previous studies have shown that the somatic PV concentration ranges from 0.8 to 70.6 μM in hippocampal dentate gyrus basket cells and from 55 to 1788 μM in cerebellar basket cells (Eggermann and Jonas, 2012). In the simulations, we have used a 5-s long depolarizing current of 100 pA and buffer concentration ranging between 0 and 1500 μM. For PV, we observe a decrease in excitability as the total buffer concentration *PV_T_* increases from 50 to 1000 μM (Figure 4A). As the buffer concentration increases from 0 to 1500 μM, the mean frequency spiking drops from 39 to 30 Hz (Figure 4B). We have observed a similar behavior for the slow and fast buffer (Figure 4B). This demonstrates that changes in the level of parvalbumin concentration changes the firing rate of the FS interneurons. This regulatory effect occurs in a similar way for the fast and slow buffers.

**Figure 5:**
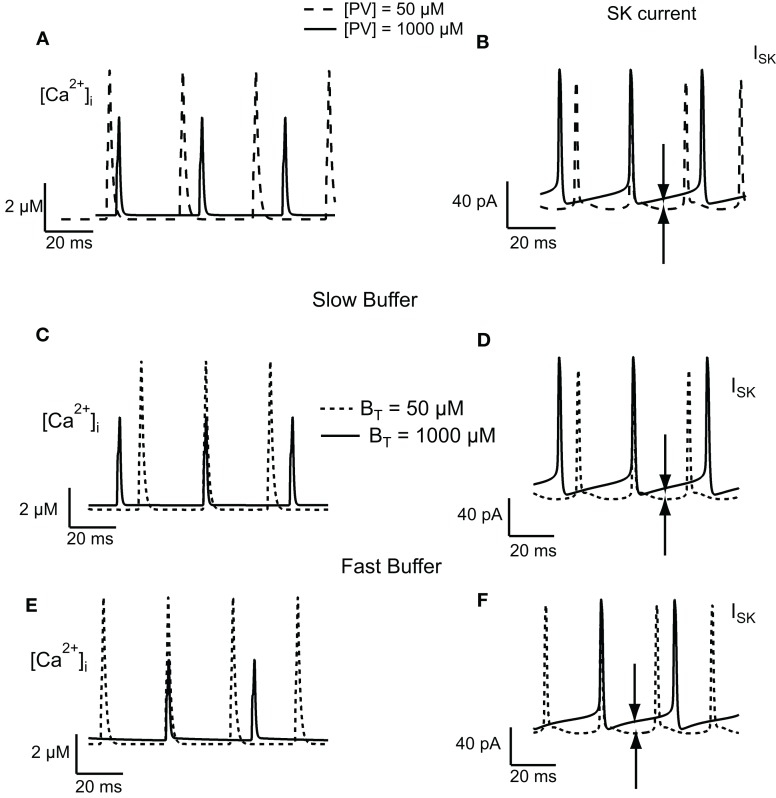
**Effect of buffer concentration on the activation of the SK current in the FS cell model**. Calcium transients obtained in response to injection of 100 pA depolarizing current for PV **(A)**, slow **(C)**, and fast **(E)** buffers. The calcium transients have a slower decay and have a higher peak value at low buffer concentration. Between two action potentials, at high buffer concentration, the residual calcium level is higher than the level at low buffer concentration. **(B,D,F)** This results in a greater activation of SK channels (indicated by the arrows in the figure), increasing the duration of the AHP, and therefore decreasing the firing frequency.

To understand the change in firing frequencies, we have investigated the effect of the buffer concentration on the activation of the SK current. We have considered the amplitude of the *I_SK_* current, the amplitude, and decay time of the *Ca*^2+^ transients. To avoid the effect of summation of *Ca*^2+^ transients, only those transients occurring at the steady-state plateau level were taken into consideration. For low PV concentrations, the decay time of the calcium transients is slower than the decay time at high PV concentration. Moreover, at low PV concentration, the amplitude of *Ca*^2+^ transients are higher than the amplitude at high PV concentration (Figure 5A). Between two AP, the intracellular calcium concentration drops to 0.1 μM (low buffer concentration, *PV_T_* = 50 μM) and 0.3 μM (high buffer concentration, *PV_T_* = 1000 μM). This value of 0.3 μM is sufficient to activate a significative fraction of the SK channels that have a *K_D_* for calcium of 300–700 nM (Hirschberg et al., 1998; Xia et al., 1998). The amplitude of the *I_SK_* current is relatively constant between two AP at low PV concentration (15 pA) while it slowly increases from 19 to 35 pA at high PV concentration (Figure 5B). This also increases the duration of the AHP and therefore reduces the firing frequency. Figures 5C–F show the results for the slow and fast buffers. As for PV, the residual calcium level between two action potentials is higher at high buffer concentration. It will activate more SK channels, increase the duration of the AHP, and decrease the firing frequency. The time course of the other ionic currents were similar when the buffer concentration was increased in the PV, slow, and fast buffering conditions (not shown).

## Discussion

In this paper, we present a new conductance-based single compartment computational model for striatal FS interneurons. Our model is adapted from the model of Erisir et al. (1999) for FS neocortical interneurons. It differs from the former model (and its modifications by Golomb et al., 2007; Ermentrout and Wechselberger, 2009) in that it includes the presence of a calcium buffer protein similar to PV. The dynamic of the calcium buffer is included in the FS model of Erisir et al. (1999) by the addition of a HVA calcium current and a SK current. Our model differs also from other FS models (Jolivet et al., 2004; Lewis and Rinzel, 2004; Mancilla et al., 2007) in that it preserves the dynamic of neurons belonging to class 2 excitability, as it is the case experimentally for FS neurons (Tateno et al., 2004).

PV is considered as a slow buffer similar to EGTA, but recent results show that it can behave like BAPTA, under physiological condition, at high concentration (Franconville et al., 2011; Eggermann and Jonas, 2012). Therefore, we have investigated the effect of PV and the effect of a calcium buffer in both cases, slow and fast binding kinetics. Our results show that calcium buffers, through modulation of the level of residual [*Ca*^2+^]*_i_* and its coupling to SK channels during a train of action potentials, control the excitability of FS interneurons. The SK current activation depends on the level of residual [*Ca*^2+^]*_i_* between AP. The residual *Ca*^2+^ concentration increases with buffer concentration, as the calcium buffer can act as a source of calcium ions during that period. Therefore the SK current between AP increases with buffer concentration leading to lower firing frequencies due to prolonged AHP. Our results show that this provides a very robust mechanism for controlling the excitability of FS interneurons. In our simulation, we have used high concentration of PV, such as 1000 μM. Such a high value of buffer concentration is exceptional and so far it has only been found in cerebellar basket cells (Eggermann and Jonas, 2012). Nevertheless our main conclusion does not rely on this specific value of PV concentration. As it is shown in Figure 4B, the firing frequency decreases as the buffer concentration increases from 0 to 1500 μM. This means that a similar decrease in the frequency of firing will be observed if the buffer concentration is raised from 10 to 50 μM, in the PV concentration range found in hippocampal basket cells (Eggermann and Jonas, 2012). Moreover, the regulation by the calcium buffer concentration appears to produce similar effects for PV, fast, and slow calcium buffers. In addition, it appears not to depend on the type of *Ca*^2+^-activated *K*^+^ conductance providing coupling between excitability and *Ca*^2+^ dynamics, as the regulation appears to be same in our FS interneuron model and in cerebellar granule cells where this effect is mediated by BK channels (Gall et al., 2003).

During a train of AP, [*Ca*^2+^]*_i_* increases due to the summation of *Ca*^2+^ transients. For sufficiently long trains of AP, [*Ca*^2+^]*_i_* will reach a steady-state plateau and fluctuate between a lower and an upper level. During the accumulation phase of *Ca*^2+^, we have observed a faster initial decay and higher amplitude of the *Ca*^2+^ transients for the slow buffer (data not shown; Markram et al., 1998). The differences in the *Ca*^2+^ transients between the slow and fast buffers attenuate at the steady-state plateau level where PV, slow, and fast buffers are already saturated. In agreement with previous published work (Helmchen et al., 1996; Lee et al., 2000) our model predicts a build-up in *Ca*^2+^ and different degrees of buffer occupancy in the three conditions (PV, slow, and fast buffers; Figures 3E,F). In our model, the summation of *Ca*^2+^ transients is responsible for the spike-frequency adaptation through the progressive activation of SK channels. Due to supra-linear summation, the fast buffer saturates rapidly whereas PV and the slow buffer lead to a slow build-up in Ca^2+^. This implies that: for trains of AP of short duration, the neuron containing a fast buffer will display spike-frequency adaptation, whereas neurons containing PV or a slow buffer will display little or no spike-frequency adaptation. Those effects will be more pronounced at a higher buffer concentration than at a lower buffer concentration (Figures 3B–D).

The purpose of our model was to propose a basic mechanism for the regulation of excitability of FS neurons by calcium buffering. Despite the use of a single compartment model with a limited set of conductances and currents, the simplicity of our model and the use of buffers with different kinetics validates our simulations to other neuronal types. Indeed, the proposed mechanism will remain valid providing that the mechanisms of excitability remain the same, as in FS neurons, and that the conductance of the *Ca*^2+^-activated *K*^+^ channels is sufficient to obtain a strong coupling between excitability and *Ca*^2+^ dynamics during the spike generation. A possible improvement in our model would be to explore the competitive binding between different calcium binding partners by considering *Ca*^2+^ diffusion, immobile, and mobile buffers (Markram et al., 1998). Parvalbumin is considered as a slow mobile buffer and SK channels form a complex with calmodulin and act as high affinity, fast *Ca*^2+^ binding partners (Stocker, 2004). Moreover, it is has been shown that in acutely dissociated CA1 hippocampal pyramidal neurons, SK channels are tightly coupled with L-type calcium channels, within a distance of 50–150 nm (Marrion and Tavalin, 1998). This spatial coupling of SK channels and *Ca*^2+^ sources promotes the formation of nano or microdomains that can modify the efficiency of calcium buffering depending on the mobility and affinity of the calcium buffers. If the *Ca*^2+^ sensor is within ~20–50 nm of the *Ca*^2+^ source, a high affinity calcium buffer like BAPTA and not the slow EGTA will be able to interfere with the *Ca*^2+^ signaling. While if the *Ca*^2+^ sensor and *Ca*^2+^ source are located in microdomains (between 50 nm and a few hundred nanometers) both BAPTA and EGTA will interfere with the *Ca*^2+^ signaling (Neher, 1998; Fakler and Adelman, 2008).

In addition to their already documented role in *Ca*^2+^ homeostasis, *Ca*^2+^-binding proteins appear to play an active role in modulating neuronal intrinsic excitability. Although, information storage is usually believed to be mediated by long-term modifications in the strength of synaptic transmission, activity-dependent changes in the neuronal intrinsic excitability also occur, causing forms of non-synaptic plasticity (Aizenman and Linden, 2000; Armano et al., 2000). Changes in the calcium buffering capacity might have an effect on this regulation. This could be the result of changes in the localization or in the level of expression of *Ca*^2+^ binding proteins. The mathematical model we present here provides a valuable tool for the investigation of the functional role of parvalbumin in the regulation of the activity of the striatal FS interneurons. This study paves the way for further theoretical work to assess the impact of calcium buffering on the activity of the striatal FS interneurons network.

## Conflict of Interest Statement

The authors declare that the research was conducted in the absence of any commercial or financial relationships that could be construed as a potential conflict of interest.
